# Examining the Landscape of Prognostic Factors and Clinical Outcomes for Cancer Control

**DOI:** 10.3390/curroncol28060432

**Published:** 2021-12-06

**Authors:** Meredith Elana Giuliani, Eleni Giannopoulos, Mary Krystyna Gospodarowicz, Michaela Broadhurst, Brian O’Sullivan, Zuzanna Tittenbrun, Sonali Johnson, James Brierley

**Affiliations:** 1 Radiation Medicine Program, Princess Margaret Cancer Centre, Toronto, ON M5G 2M9, Canada; Mary.Gospodarowicz@rmp.uhn.ca (M.K.G.); Brian.OSullivan@rmp.uhn.ca (B.O.); James.Brierley@rmp.uhn.ca (J.B.); 2 Department of Radiation Oncology, University of Toronto, Toronto, ON M5T 1P5, Canada; 3 Department of Cancer Education, Princess Margaret Cancer Centre, Toronto, ON M5G 2N2, Canada; eleni.giannopoulos@uhnresearch.ca (E.G.); Michaela.Broadhurst@uhnresearch.ca (M.B.); 4 Knowledge, Advocacy and Policy, Union for International Cancer Control, 1202 Geneva, Switzerland; tittenbrun@uicc.org (Z.T.); johnson@uicc.org (S.J.)

**Keywords:** prognostic factors, clinical outcomes, cancer control

## Abstract

Prognostic factors have important utility in various aspects of cancer surveillance, including research, patient care, and cancer control programmes. Nevertheless, there is heterogeneity in the collection of prognostic factors and outcomes data globally. This study aimed to investigate perspectives on the utility and application of prognostic factors and clinical outcomes in cancer control programmes. A qualitative phenomenology approach using expert interviews was taken to derive a rich description of the current state and future outlook of cancer prognostic factors and clinical outcomes. Individuals with expertise in this work and from various regions and institutions were invited to take part in one-on-one semi-structured interviews. Four areas related to infrastructure and funding challenges were identified by participants, including (1) data collection and access; (2) variability in data reporting, coding, and definitions; (3) limited coordination among databases; and (4) conceptualization and prioritization of meaningful prognostic factors and outcomes. Two areas were identified regarding important future priorities for cancer control: (1) global investment and intention in cancer surveillance and (2) data governance and exchange globally. Participants emphasized the need for better global collection of prognostic factors and clinical outcomes data and support for standardized data collection and data exchange practices by cancer registries.

## 1. Introduction

Prognosis is commonly referred to as a “probability or risk of an individual developing a particular state of health (an outcome) over a specific time, based on the clinical and non-clinical profile” [1]. Prognostic factors in cancer may be used to determine certain outcomes [1,2], and numerous factors can influence the clinical outcome, including the tumour profile, the anatomic disease extent, the patient characteristics—with co-morbidities and treatments—and the social determinants of health [3,4]. As such, treatment decisions depend on the complex interplay between disease-related factors (e.g., extent of disease), patient factors, as well as treatment risks, efficacy, and toxicity [5]. Moreover, the variety of outcomes and their independence over time requires a complex algorithm to capture the impact of interventions that are available to ameliorate the adverse impact in the present or the past. Thus, while prognostication remains crucial in various aspects of care—including treatment decisions and advanced-care planning [6]—accurate prognostication—including appropriate formulation and precise communication about prognosis—remains a significant challenge for healthcare providers and research groups globally [7].

Cancer represents a heterogeneous group of diseases, and patient diversity adds to this heterogeneity. Efforts to develop a standard set of prognostic measures to improve clinical predictions for cancer remains a significant challenge [8]. Collaborative efforts between the American Joint Commission on Cancer (AJCC) and the Union for International Cancer Control (UICC) as well as evidence from population-based patient cohorts have led to the development of cancer-staging classification that have prognostic value and provide information to refine treatment [9]. Currently, no standardized general framework for non-anatomic prognostic factors has been adopted. Data items related to cancer stage, treatment, and prognosis have been integrated into various cancer surveillance programs, including cancer registries, in order to facilitate and guide data collection [10]. However, national and regional cancer registries may vary with respect to the types of data items collected [11].

Understanding cancer prognosis can facilitate patient comparisons, appropriate clinical trial design and analysis, policy making, and cancer control programs [12,13,14]. At a population level, an understanding of prognosis can contribute to identification of individuals at greater risk of cancer mortality, thereby supporting prevention efforts as well as the monitoring of the results of cancer control interventions. Consequently, these factors should be collected by cancer registries to enhance cancer control and promote appropriate care. We sought to explore the perceptions surrounding the collection and application of prognostic factors and clinical outcomes data to understand their utility, function, and standardization in cancer control settings. 

## 2. Materials and Methods

### 2.1. Study Design

This study employed a qualitative design using in-depth, one-on-one interviews with international representatives involved in cancer registries and various cancer control organizations. The University Health Network Research Ethics Board provided ethical exemption for the study.

### 2.2. Data Collection

#### 2.2.1. Sampling and Recruitment of Study Participants

A purposive sampling strategy was used to select individuals from various stakeholders in diverse geographic regions (e.g., high-, upper-middle-, lower-middle-, and low-income regions). Participants came from a variety of disciplines, including medical and surgical oncology, as well as clinical and health services research and leadership in major cancer control activities.

Participants were invited to take part in one-on-one, semi-structured interviews and were selected based on their expertise in the area of population-based outcomes in cancer, cancer surveillance, and cancer control. Each was sent an e-mail invitation containing a description of the study, and all agreed to take part. Snowball sampling was also used, whereby participants recommended additional individuals who would be interested in taking part in interviews [15]. Recruitment continued until thematic saturation was achieved.

#### 2.2.2. Interview Procedure

Participants were recruited to participate in one-on-one, semi-structured interviews that took place between June and September 2020. The duration of the interviews averaged thirty minutes and ranged from twenty minutes to one hour in length. Interviews were conducted via telephone or through electronic interfaces, such as Skype.

The study team developed an interview guide (See Supplementary Materials) to ascertain participants’ perceptions surrounding key challenges and considerations regarding the collection of prognostic factors and outcomes data in the context of global cancer control. Questions for the interview guide were developed based on consensus discussions from a virtual workshop held by the UICC in May 2020 and feedback provided by members of the study team. 

Additionally, an inductive approach was used to gain an understanding of participants’ experience, and probes were used to elicit further details. Participants were asked about their engagement in cancer prognosis and staging and their familiarity with these subjects. Subsequently, participants were asked to describe (1) what they know about the scope of the factors commonly collected in cancer registries; (2) describe which factors are most important; and (3) describe challenges or barriers to collecting prognostic factors and outcomes data (see Supplementary Materials). 

A trained research assistant (MB) conducted interviews with study participants. Interview participants were also asked to reflect on the current climate and trends in the data collection and expectations of how these may evolve in the future. All interviews were audiotaped, de-identified, and transcribed verbatim. 

### 2.3. Data Analysis

The study investigator (MEG) coded interview transcripts using an inductive thematic analysis approach, which was based on principles of phenomenology. This approach focuses on understanding individual experiences and interpretations of lived events [16]. Patterns observed in the data are first identified and are subsequently organized into meaningful themes. Additionally, a framework developed by the authors was used to guide categorization of prognostic factors and cancer outcomes described by participants and to support data interpretation [17]. 

Following coding of the interview transcripts, an independent second reader and coder (EG) was consulted for collaborative analysis to enhance comprehensiveness and to improve credibility. Discrepancies in coding were discussed until consensus was reached. A constant comparative and iterative approach was used. Data were collected until thematic sufficiency was attained [18]. NVivo version 11 software was used to assist with coding and analysis.

## 3. Results

### 3.1. Participant Characteristics

Ten individuals, including those working in cancer registries and experts in global cancer control research, were invited to participate in semi-structured interviews. The majority of participants were male (80%). Participants self-reported their role, location, and work in cancer control. Participants came from clinical and non-clinical backgrounds and included clinicians (surgeons and pathologists), statisticians, healthcare executives, and scientists whose work spanned distinct geographic regions. Five participants were located in North America, four in Europe, and one in Asia. The majority of participants (80%) worked in middle-high-income countries. 

### 3.2. Scope of Currently Collected Prognostic Factors and Outcomes

Participants were asked to describe prognostic factors and cancer outcomes that should be collected by cancer control organizations and population-based registries in an ideal situation. A summary of prognostic factors as reported by participants is summarized in Table 1. 

All respondents felt that the anatomic extent of disease was the most important data element to be captured. In terms of population-based outcomes, the majority agreed that mortality data is already collected comprehensively, and future efforts should focus on other areas, including cancer recurrence, patient-reported outcomes, and quality of life. 

Participants with expertise in cancer control research discussed standardizing and prioritizing collection of cancer recurrence data, while participants with expertise in health services research discussed prioritizing collection of patient-related demographics, such as social determinants of health data, patient-reported outcome (PROs), and quality of life (QOL) and performance status.

### 3.3. Infrstructure and Funding Challenges

**Figure 1:**
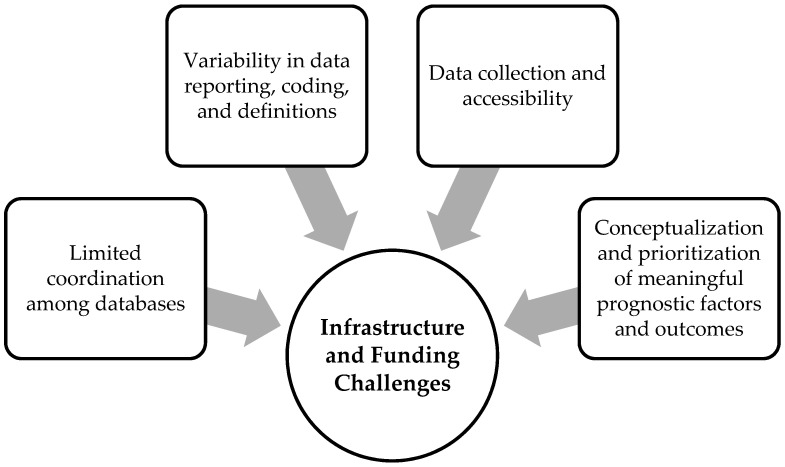
Four identified barriers related to infrastructure and funding challenges, including data collection and access; variability in data reporting, coding, and definitions; limited coordination among databases; and conceptualization and prioritization of meaningful prognostic factors and outcomes.

Participants described several barriers related to infrastructure and funding challenges that affect the collection of prognostic factors and outcomes. These include data collection and accessibility; limited coordination among databases; variability in data coding, reporting, and definitions; and conceptualization and prioritization of meaningful prognostic factors (Figure 1).

#### 3.3.1. Data Collection and Accessibility

Many participants perceived a lack of cancer registration and timely access to registry data as a barrier to data collection as well as implementation of cancer surveillance activities: “The lack of cancer registries is a consistent and repeated problem. It’s certainly improving over time, but we don’t have what we need, particularly in low- and middle- income countries, without question, in order to be able to get the sort of data that we require” (P04).

P08 further noted the challenge related to the collection of prognostic factors data in various parts of the world: “It’s really difficult for a registry to collect that. I think some clinical registers in higher-income countries can do that. But for the bulk of registries in most of the world, I think it is very difficult to collect the majority of these factors that are now listed in these prognostic factors in the manuals, for example.” 

Some discussed the challenge of unfeasible data collection methods and related implications for data acquisition in different regions and jurisdictions. Accordingly, one participant (P03) noted that “Just one-third of centers had any form of electronic medical record as a way of data capture—two-thirds of centers still rely on paper and pen, which means that they don’t lend themselves very easily to collation of data.” 

Four participants expressed concerns regarding limited access to registry data and felt that cancer registry data should be made available for use publicly: “It is very difficult to get this data, and we should have the opportunity to access this data. Of course, data cleaning and analysis takes a huge amount of time—and researchers who do this work need to ensure that they realize the benefits and the fruits of their labours—but the raw data could be made available. That will then encourage researchers to use the data” (P10).

#### 3.3.2. Limited Coordination among Databases

Many participants described the issue of a lack of data coordination. P02 noted that “In many countries, care is incredibly fragmented between the private and the public healthcare system…there are numerous payers, providers, and institutions involved. I think that in in low- and middle-income countries, that’s probably the biggest barrier—is getting data across all these different players in the healthcare system.”

Some participants discussed various challenges related to data sharing and exchange in research, including a lack of willingness or incentives to share prognostic factors and outcomes data, data elements heterogeneity in what is collected, and definitions used and lack of collaboration among various groups: “The approach to research and data repository is much more individualistic and institutionally based in North America and less collaborative” (P01). Some suggested that these factors may by reflected by differences in funding systems across healthcare institutions. 

#### 3.3.3. Variability in Data Reporting, Coding, and Definitions

All participants reflected on the challenge in dealing with heterogeneous datasets. As P08 noted, “It’s (data) not collected in a systematic way.” While there are data standards for coding, many participants noted inconsistencies in how prognostic factors and outcomes are collected, the extent to which these factors are collected, as well as the criteria used to define them. Participant 10 described the nuance in terminologies used by clinicians versus researchers: “Recently, I had a conversation with a colleague, and we were talking about survival. I was talking about five-year net (cancer specific) survival, but my other colleague was talking about overall survival, and these are different. Another example is in relation to social determinants; there are fairly precise definitions, which are used differently by different research groups…differences in the way we use these terms exist.”

Many participants also reflected on issues related to inconsistent data reporting and dealing with missing data. Participants discussed the increasing use of data modeling in many parts of the world, and many felt it was important to acknowledge the challenges related to the use of extrapolated data: “The extrapolation and modeling is not the reality of many countries” (P09). As P02 noted: “It makes it very difficult to extrapolate one data set to another jurisdiction. So, internally within a given country, it makes it difficult to understand if your results are real. And then, if we’re looking to compare outcomes and treatments across different countries, if they’re collecting data in a different way or defining things differently, then it makes it very difficult and perhaps dangerous to begin comparing outcomes that are different.”

#### 3.3.4. Conceptualization and Prioritization of Meaningful Prognostic Factors and Outcomes

Participants described the challenge in conceptualizing and prioritizing which prognostic factors are most meaningful. P05 noted that it was difficult to know: “how to go about the (collection of prognostic factors)” and described a “lack of appreciation and lack of resources at the disposal of some registries, given the infrastructure of the health system and the part of the world”. 

The majority of participants discussed the lack of prioritization on collecting patient factors: “We don’t have all the information on the patients, for example, co-morbidities, all these other factors that influence outcomes” (P08). Another participant noted: “There are clinical outcomes, plus societal outcomes, and patient-reported outcomes. These may relate to functionality—outcomes that patients value beyond the clinical outcome” (P10). However, many acknowledged the limited capacity of cancer registries to collect large amounts of data, which makes it difficult to determine what is most important. 

Some participants perceived that varying interests, e.g., by researchers, registries, and clinicians, contribute to the difficulty in prioritizing data collection and felt it was important to promote greater collaboration and discussion among these groups (e.g., researchers, clinicians, cancer registries, and cancer control organizations) to determine their interests. As P06 noted: “So much of what is published in medical literature cannot be translated easily into the WHO blue books (i.e., tumour pathology classification) or UICC classification. And the reason it’s a big task is partly incentives of different groups.”

### 3.4. Future Priorities for Cancer Control

The majority of participants described two priority areas for future cancer control efforts, including investment and intention in cancer surveillance and data governance and exchange globally.

#### 3.4.1. Global Investment and Intention in Cancer Surveillance

All participants discussed the importance of prognostic factors and outcomes data for policies, funding, public health initiatives, and treatment decisions: “I think it’s incredibly important to have this information because it does impact on policy makers, impacts clinicians, it drives change. I’m speaking specifically on one or two metrics, but generally having this information and better understanding what is driving differences between different worlds regions, different populations, subpopulations is incredibly important. If you can present this to the stakeholders, you can certainly have an impact” (P05). The participant further described the importance of leadership and championing efforts by clinicians: “We do international benchmarking, but we want the capacity build so that the registries actually become more important to the clinicians. Influential clinicians are (essential for) sustainable development of the cancer registry. And if that can be linked to change government practice, or at least recommendations that are implemented nationally, then that’s what we want to see.” 

However, many also discussed how funding and policy decisions depend on the health system context and degree of data collection. One participant described the approach to data collection as a “tiered” system: “As a system becomes more sophisticated, you can collect more sophisticated data… the method of collecting that’s useful for changing policy belongs to the most evolved systems” (P09). The majority of participants described two priority areas for future cancer control efforts, including investment and intention in cancer surveillance and data governance and exchange globally.

#### 3.4.2. Data Governance and Exchange Globally

Participants discussed the importance of global data governance as a priority area and a need for data governance technologies that can enable appropriate exchange of health information. Many also felt it was important for cancer control organizations and cancer registries in different regions to create a core, merged dataset that may support standardized data collection globally (e.g., pathological and clinical stage globally). 

Participant 03 noted that “One opportunity in the digital era is to try and create datasets that bring together data from multiple sources, and in many countries, that should be possible.” Further, participants suggested combining different naming systems to promote streamlined exchange and uniform use of prognostic factors and outcomes data: “We can harmonize the definitions, what people actually collect, and I think we can do a lot with the International Collaboration on Cancer Reporting (ICCR). So, I think the combination of the three groups—ICCR, UICC, AJCC as well, (because of staging, and they are really thinking about the molecular side of things, too). I think it would be something that is doable, and I would probably go for the common tumors first” (P06).

## 4. Discussion

This study sought to explore perceptions about the current use and application of prognostic factors and clinical outcomes in supporting global cancer control efforts. Participants described the anatomic disease extent as being the most important and consistent factor that influences cancer prognosis and is categorized based on the TNM staging classification [19,20,21]. This finding is supported in the literature [22,23]. Additionally, participants suggested prioritizing the collection of outcomes, such as cancer recurrence, patient-reported outcome measures (PROs), and quality of life (QOL), rather than mortality data, which are already comprehensively collected in most regions. Many registries do not routinely document cancer recurrences [24,25], and findings from studies aiming to address this by using specific diagnostic codes have not been reliable or generalizable. Examining survival alone is not sufficient for comparing treatment outcomes and decision making. Further, cancer recurrence data is important for large population-based research [26].

Evidence also suggests that while there has been increasing interest in collecting PROs, large-scale implementation has been slow due to limited resources, hesitance by clinicians, concerns about how data is used, and technological issues [27,28]. In a study examining implementation of a widespread PRO program in an integrated health system, increased clinician engagement, payer incentives, and clinical championing efforts were associated with improved collection of PROs [28]. Findings from the present study suggest that participants supported the collection of PROs in addressing fundamental patient outcomes, such as health-related QOL and patient satisfaction [29]. Use of PROs have also been shown to result in increased overall survival for metastatic cancers [30]. Overall, these findings add to the existing literature on the value of patient outcomes as particularly salient clinical endpoints and measures of quality of care. 

Participants described the scope of data collection practices (e.g., data collection and accessibility, variability in data reporting, coding and definitions, lack of coordination between cancer registries and data prioritization) as a barrier to uniform global data collection and coding due to differences across regions, institutions, and needs from various health and funding bodies. This heterogeneity in data practices therefore has a direct impact on collaborative research efforts, patient care, and policy decisions regarding prevention, diagnosis, and treatment and impedes international efforts towards standardisation of data collection to support global cancer surveillance and control. The literature has previously cited data ownership, privacy, and security standards as factors that influence the level of data access and distribution [31,32]. Restrictions in data processing may affect researchers and registries’ ability to conduct precise population-based analyses and offer sound evidence regarding risk factors and overall public health [33]. In line with this, participants raised concerns about the issues with data modelling and alluded to the consequences of using poor or incomplete data to highlight different cancer outcomes and drive policy decisions. For instance, in many low- and middle-income countries, surrogate mortality data are often used to describe the cancer incidence (e.g., cancers with poor survival proportions). While data on death is important for informing public health priorities, it must be examined together with trends in cancer incidence to understand the true effect of cancer within a population [34]. There is a need for researchers, oncologists, and various health care bodies to support and advocate for improved data access, sharing, and completeness such that researchers are able to use this data, and registries are able to highlight meaningful, population-level differences [35].

One key finding was regarding inconsistent use of definitions, reporting, and coding of data as being a barrier to data interpretation. Cancer registration varies with respect to the type of data collected and the level of detail. Variability in data reporting across jurisdictions may affect how cancers are classified and clinical decisions that are made. Heterogeneous terminologies used in hospitals and healthcare facilities ultimately prevent correct reuse of the data by cancer registries [33]. Collected data are derived from various hospitals and institutions and are tracked by cancer registries to describe different clinical outcomes, such as mortality or response to treatment. Concomitant use of data from these different health bodies presents a challenge in terms of cancer monitoring [36]. There has been support for the use of harmonized nomenclatures to prevent ambiguity in registration practices [37], pathology reporting [38], and epidemiological research [39]. One study found a 7.3% difference between unadjusted and adjusted survival due to differences in international registration practices [40]. Another study describing a terminology used for reporting in cancer clinical trials found evidence of incorrect data interpretation and reporting, which may have led to false clinical decisions [41]. The current findings support the need for data standardization by combining various terminologies and integrating them into practical, user-friendly systems that can be used widely. This in turn would reflect better global registration practices, improve data uniformity. and prevent biased results. In the context of health policy, having accurate information about prognosis or expected outcomes is important for prioritizing resource utilization in different populations [42]. However, without application of a consistent and widespread terminology, analyses may be inaccurate, and decisions may not be appropriate.

This study contributes to the literature, as it describes important barriers and future considerations related to the collection of prognostic factors and outcomes data. These findings demonstrate that appropriate data governance, exchange, and global investment in cancer surveillance are needed to advocate for better collection. Further, streamlined exchange of data by cancer registries (e.g., hospital and population based) can allow researchers to engage in comparative effectiveness research and inform decisions based on interventions implemented in different health contexts [43]. Overall, these findings can help inform strategic planning of cancer control programmes and offer considerations for health bodies wishing to expand collection of their data. Additionally, addressing the shortcomings of current processes and governance is as important as expanding what is collected.

Results from this study highlight the importance of cancer registration. Future work will focus on evaluating various types of cancer registries (e.g., hospital based and national) in low-, middle-, and high-income countries and identifying how collected data is used by researchers, public health groups, clinicians, and statisticians. 

This study has some limitations. First, interviews were conducted with English-speaking participants only, and it was not the focus of this work to explore the patient perspective. This valuable area requires a dedicated study. Secondly, the use of snowball sampling may have inadvertently biased the study results. Third, the majority of current activities, including data collection through registries and publications from such data sets, are from high-middle-income countries. The interviews emphasize the need for investment and prioritization of data collection efforts globally. This study explored, at a high level, current prognostic factors and outcomes in cancer control, and as such, the inventory was not meant to be comprehensive. 

## 5. Conclusions

Collection of prognostic factors and clinical outcomes has important utility in patient are, research, and health policy. Focusing efforts on patient-reported outcomes and addressing barriers, such as data accessibility, coordination, prioritization, and variability, are important for supporting standardized cancer registration. Additionally, ongoing investment in cancer surveillance programs and better data governance will be important for improving practices over time.

## Figures and Tables

**Table 1:** Prognostic factors and clinical outcomes collected in an ideal situation.

Prognostic Factors and Outcomes	Category	Data Points Reported by Participants	Representative Quotes
Disease Characteristics	Tumour Characteristics	Grade Differentiation Molecular markers	“…Standardizing the quality of pathology reporting would be part of this exercise because if you don’t get reliable pathology reporting, like grade or degree of differentiation and so on, you’re likely to end up with inaccurate information.” (P03)
	Anatomic disease extent	Stage of presentation	“…it’s very difficult to understand outcomes if you can’t adjust for the stage or understand the stage (at presentation). I think stage is a surrogate for access challenges in low- and middle-income countries—those can be geographic, financial, and cultural” (P01)
Host-related prognostic factors	Demographics	Sociodemographic factors (e.g., age, education, socioeconomic status)	“…we don’t collect ethnicity data routinely in our health data sets, and it’s very challenging to understand structural inequalities in health care, access, and uptake as a result of the ethnic and sociocultural variables if you don’t collect that data.” (P01)
	Co-morbidities	Robust marker of co-morbidity Performance status Smoking status BMI Alcohol consumption	“In an ideal world, we would have a robust marker of co-morbidity…co-morbidity would be very problematic and probably lack uniformity” (P02) “We could probably include five or ten different things across all cancers that would be really important to have, like smoking status, body mass index, alcohol exposure.” (P02)
Environment-related prognostic factors & Social Determinants of Health	Access to treatment	Quality of care received (e.g., reasons for non-treatment) Specific issues of access to care: Geographic access, financial access, and cost data	“…how a patient pays at the point of care—do they have to pay out of pocket, is there a government-funded insurance scheme, a social insurance scheme, is it private insurance, or is there a tax payer funded health system that includes cancer care?” (P01) “…something that is often missing is reasons for non-treatment. Is it because they were not referred? Is it because they saw the doctor, and it was not recommended, or is it because it was recommended, and the patient elected not to have it, and if so, what was the reason? Fear, personal preference values, financial toxicity?” (P02)
	Quality of Care	Income level Education Health literacy	
Outcomes	Disease Outcomes	Mortality Recurrence/relapse Cancer-specific survival Local disease control	“We’re really interested in binary outcomes. Does the patient survive and for how long?” (P08) “In an ideal world, date of relapse, some patient-reported outcomes, quality of life data. That would be the core group of outcomes.” (P03)
	Quality of Life Outcomes	Quality of Life Patient-reported outcomes Toxicity	

## Data Availability

Data from the study are available by contacting the authors.

## Supplementary Materials

The following are available online at https://www.mdpi.com/article/10.3390/curroncol28060432/s1, Supplementary Materials: Interview Guide.
