# Supplementary material for: Examining the Landscape of Prognostic Factors and Clinical Outcomes for Cancer Control
Source: Curr Oncol. 2021 Dec 6;28(6):5155–66. doi: 10.3390/curroncol28060432 (PMC8699872; doi:10.3390/curroncol28060432)
Supplement: Supplementary file 1 [file curroncol-28-00432-s001.zip › curroncol-1450320-supplementary.pdf]

SUPPLEMENTARY MATERIALS

Introduction

1. To begin, could you please provide us with some background about yourself:
    a. What is your current role?
    b. How long you have been involved with cancer control activities / prognostic factors work / health systems work?

Cancer Prognostic Factors

2. What is your understanding of the current collection of prognostic factors in cancer?
3. What prognostic factors does your organization currently collect?
    a. Without any financial or logistical limitations, what other prognostic factors should ideally be collected?
4. How do you define or decide what prognostic factors need to be collected for population-based cancer registries?
    a. What is the process for making these decisions at your organization?
5. What do you perceive to be the biggest the barriers in the collection of prognostic factors?
    a. Outside of financial constraints?

Cancer Outcomes

6. What is your understanding of the current collection of outcomes data in cancer?
7. What clinical cancer outcomes does your organization currently collect?
    a. Other than survival / death?
    b. Without any financial or logistical limitations, what other clinical outcomes should ideally be collected?
8. How do you define or decide what outcomes should be collected for population-based cancer registries?
    a. What is your process for making these decisions?
9. What are the barriers to collection of cancer outcomes?
    a. Outside of financial constraints?

Applications/Future Outlook

10. With respect to both prognostic factors and / or clinical outcomes, once this data is collected how is it being used?
    a. How do you expect the application of this data to evolve in the future?
11. What is needed to move us forward in the collection of prognostic factors and outcomes?
